# A high-density SNP genetic linkage map for the silver-lipped pearl oyster, *Pinctada maxima*: a valuable resource for gene localisation and marker-assisted selection

**DOI:** 10.1186/1471-2164-14-810

**Published:** 2013-11-20

**Authors:** David B Jones, Dean R Jerry, Mehar S Khatkar, Herman W Raadsma, Kyall R Zenger

**Affiliations:** Centre for Sustainable Tropical Fisheries & Aquaculture, The School of Marine and Tropical Biology, James Cook University, Townsville, QLD Australia; Reprogen - Animal Bioscience, Faculty of Veterinary Science, University of Sydney, Camden, NSW Australia

## Abstract

**Background:**

The silver-lipped pearl oyster, *Pinctada maxima*, is an important tropical aquaculture species extensively farmed for the highly sought "South Sea" pearls. Traditional breeding programs have been initiated for this species in order to select for improved pearl quality, but many economic traits under selection are complex, polygenic and confounded with environmental factors, limiting the accuracy of selection. The incorporation of a marker-assisted selection (MAS) breeding approach would greatly benefit pearl breeding programs by allowing the direct selection of genes responsible for pearl quality. However, before MAS can be incorporated, substantial genomic resources such as genetic linkage maps need to be generated. The construction of a high-density genetic linkage map for *P. maxima* is not only essential for unravelling the genomic architecture of complex pearl quality traits, but also provides indispensable information on the genome structure of pearl oysters.

**Results:**

A total of 1,189 informative genome-wide single nucleotide polymorphisms (SNPs) were incorporated into linkage map construction. The final linkage map consisted of 887 SNPs in 14 linkage groups, spans a total genetic distance of 831.7 centimorgans (cM), and covers an estimated 96% of the *P. maxima* genome. Assessment of sex-specific recombination across all linkage groups revealed limited overall heterochiasmy between the sexes (i.e. 1.15:1 F/M map length ratio). However, there were pronounced localised differences throughout the linkage groups, whereby male recombination was suppressed near the centromeres compared to female recombination, but inflated towards telomeric regions. Mean values of *LD* for adjacent SNP pairs suggest that a higher density of markers will be required for powerful genome-wide association studies. Finally, numerous nacre biomineralization genes were localised providing novel positional information for these genes.

**Conclusions:**

This high-density SNP genetic map is the first comprehensive linkage map for any pearl oyster species. It provides an essential genomic tool facilitating studies investigating the genomic architecture of complex trait variation and identifying quantitative trait loci for economically important traits useful in genetic selection programs within the *P. maxima* pearling industry. Furthermore, this map provides a foundation for further research aiming to improve our understanding of the dynamic process of biomineralization, and pearl oyster evolution and synteny.

**Electronic supplementary material:**

The online version of this article (doi:10.1186/1471-2164-14-810) contains supplementary material, which is available to authorized users.

## Background

The silver-lipped pearl oyster, *Pinctada maxima*, is an important tropical aquaculture species that, along with *P. margaritifera*, produces almost 50% of marketed pearls worldwide by value [1]. However, like most aquaculture industries, pearl culture is still in its infancy compared to terrestrial animal production systems and has yet to establish advanced selective breeding programs required for industry advancement. Profitability of the *P. maxima* industry is driven primarily by the grading of the five pearl quality traits: shape, size, colour, lustre and surface complexion. Large variation is observed during harvest for each of these traits, presenting the potential to increase industry profitability through selective breeding. Although traditional animal improvement methods have had some success improving traits which are easy to measure in candidates under selection (i.e. animal growth) [2], they are not particularly effective for complex pearl quality traits, which are generally hard to measure, expressed late in life and generally have low heritability [3, 4]. Promising developments in livestock genomics are opening up opportunities, allowing genomic information to be incorporated into breeding programs in order to increase the rate of genetic gain for complex commercial traits in oyster. The current impediment to the implementation of genomic approaches in mollusc breeding programs, however, is a significant lack of genomic resources such as genome-wide molecular markers, genomic maps and genome sequences [2, 5, 6].

A robust high-density genetic linkage map for *P. maxima* is a fundamental precursor to understanding the architecture and evolution of pearl oyster genomes, determining the genetic basis of complex phenotypic traits under natural and industrial settings, and identifying genes and quantitative trait loci (QTL) associated with bivalve shell biomineralization. Such resources are invaluable for the development and incorporation of marker-assisted selection (MAS) into breeding programs aiming to fast track improvements in pearl quality. Presently, no genetic maps are available for *P. maxima*, with information on this species’ genome largely limited to the general physical description of its chromosomes (i.e. *N* = 14, 10 submeta- or metacentric, and four telocentric chromosomes) [7].

Preliminary genetic linkage maps have been developed for only a few bivalves, including the edible oysters *Crassostrea virginica*[8], *C. gigas*[9, 10], *Ostrea edulis*[11] and one pearl oyster species, *Pinctada fucata martensii*[12]. However, information from these maps is of limited use in *P. maxima* for molecular breeding studies, as they either consist of non-transferable markers [i.e. amplified fragment length polymorphisms (AFLPs)], have low marker density (100–200 markers), or the original species is phylogenetically too distant to be useful in a comparative genetic mapping approach [13].

Alongside the lack of genomic resources, several fundamental aspects of pearl oyster biology still remain unclear. For example, one of the most striking features of pearl oysters is that they are non-obligatory protandrous hermaphrodites (i.e. mature first as males and later change to females). In *P. margaritifera* for instance, individuals develop as males and remain so for the first two years of life before progressively changing to females reaching a sex ratio close to 1:1 at around 8 years old [14]. Sex change is known to be largely driven by environmental factors such as stress. However, the genetic determinates of this unusual life history have yet to be investigated in detail. Genetic linkage maps may be implemented to unravel some of the genetic determinates of sex differentiation and sex change in oysters. Linkage maps are also highly desirable for evolutionary genetic research and comparative mapping which would improve our understanding of pearl oyster chromosome evolution and help identify homologous chromosomal segments involved in the genetic control of economical and adaptive traits for species in the genus *Pinctada*.

This study aimed to construct medium to high density sex-average and sex-specific genetic linkage maps for the silver-lipped pearl oyster, *P. maxima*, by utilising a recently developed single nucleotide polymorphism (SNP) array [15]. Following robust linkage map construction, this study evaluates heterochiasmy between the sexes, extent of linkage disequilibrium (LD) across the genome, and the localization of important biomineralization genes. This comprehensive genetic resource allows for the first time the ability to obtain new insights into the biological and genomic architecture of this important marine species, including the identification of the genetic basis of complex phenotypic traits.

## Methods

### Reference mapping families and DNA extraction

**Figure 1:**
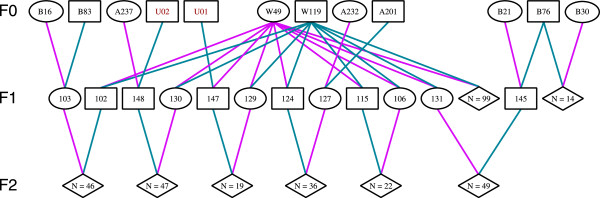
**Schematic representation of reference mapping families.** Ovals represent females, squares represent males and diamonds represent families consisting of *N* offspring of unknown sex. Pink lines show the maternal contribution to the subsequent generation and blue lines show the paternal contribution. The population of origin for F_0_ oysters is indicated by the letter in the sample ID: B for Bali, A for Aru and W for West Papua. The two unknown sires with no genotypes, U01 and U02, are indicated in red text.

To provide sufficient resolution for mapping dense numbers of genetic markers, a large mapping resource consisting of 335 individuals belonging to six phase known (3 generation) and two phase unknown (2 generation) families was generated. All families were founded by individuals collected from three genetically distinct populations (Bali, 8.32’S, 114.92’E; Aru, 6.43’S, 134.63’E; and West Papua, 1.13’N, 130.54’E). To obtain this mapping resource, numerous families were reared and bred between 2008 and 2010 at two Indonesian commercial sites (Bali and Lombok) by Atlas South Sea Pearl Ltd. see [3]. All experimental animal research was performed in accordance with James Cook University’s requirements and guidelines. To ensure only the most informative families were retained for genetic mapping purposes, genetic relatedness and diversity indices of all available F_0_ and F_1_ parents were evaluated using a set of six microsatellite markers see [3] and the most informative parent pairs were selected for breeding (relatedness values calculated in KINGROUP [16]). In total, these families consisted of 219 F_2_ progeny, 118 F_1_’s and 14 F_0_’s and the number of offspring per family ranged from 14 – 99 (Figure 1). Seven of the eight families shared common grandparents and there were two unknown grandsires as indicated by the sample IDs U01 and U02. Unknown grandsires were validated using half-sib clustering algorithms executed in Colony version 2.0 [17], but inferred genotypes were not used in map construction. Schematic representations of the pedigrees were drawn with Pedigraph Version 2.4 [18]. High quality genomic DNA was extracted from all 351 oysters using a modified CTAB protocol [19]. DNA quality was determined by agarose gel electrophoresis and each samples’ concentration was standardised to 50 ng/uL using PicoGreen dsDNA quantification (Invitrogen).

### SNP selection, genotyping and data integrity

A total of 1,189 informative *P. maxima* type I SNP markers (developed and validated in [15]) were selected for linkage map construction. Strict data integrity measures were implemented to ensure that only the most accurate SNPs were included in the analysis, as even a small proportion of genetic marker errors can dramatically affect the accuracy of genetic linkage maps [20]. Briefly, SNPs were selected if they amplified successfully, returned clear genotype calling clusters, had a minor allele frequency (MAF) > 0.01, did not deviate from Hardy-Weinberg equilibrium (*P* value cut off), conformed to Mendelian inheritance (MI) patterns from parent to offspring, did not exhibit duplicated SNP clustering patterns and had a call rate > 90%. Of the available 1,189 SNPs, 1,167 were informative for the subset of 351 oysters belonging to the mapping families and included in linkage map analysis (Additional file 1). All SNPs have previously been annotated with gene identity and Gene Ontology (GO) terms as described in Jones et al. [15].

### Map construction and genome coverage

To generate the most accurate genetic linkage maps two versions of CRI-MAP [21] were employed. Firstly, a modified version of CRI-MAP developed by Liu and Grosz [22] was utilised to calculate sex-average and sex-specific two-point recombination rates and logarithm of the odds (LOD) scores for all pairs of markers. Linkage groups (LGs) were then identified using AUTOGROUP [22] by identifying markers that co-segregate with pairwise LOD scores > 5. AUTOGROUP utilises an iterative process with a succession of parameters decreasing in stringency through five layers to ensure accurate groups are produced. Each layer consists of the following four parameters; the minimum threshold of LOD scores for linkage to be included, the minimum number of informative meiosis for a marker to be included, the maximum number of linkage groups a marker is allowed for having linkages, and the minimum threshold for the linkage ratio to which a marker shows qualified links to the best linkage group. By layer, the parameters were a) layer one: 100, 2.0, 2, 0.9, b) layer two: 50, 1.5, 5, 0.8, c) layer three: 20, 1.0, 8, 0.7, d) layer four 10, 0.5, 10, 0.6, and e) layer five: 5, 0.1, 15, 0.5. Layer five defines the cut off for a marker to be included in a linkage group. Linkage groups were numbered in order of decreasing number of markers placed within each linkage group during the AUTOGROUP phase. Once linkage groups of markers were established, predefined haplogroups of tightly linked loci (i.e. LOD ≥ 3.0 and theta ≤ 0.03) were identified using HAPLOGROUP. This produced a subset of primary (most informative) markers from each haplogroup that were selected for initial construction of a framework map.

The BUILD and FLIPS commands of the second version of CRI-MAP (2.503) modified by Jill Maddox and Ian Evans (unpublished data) were utilised to determine the marker order within each linkage group as it has been designed to deal with large datasets more efficiently. The linkage mapping strategy consisted of a hierarchal approach whereby markers were included if they could be assigned a position over the next most likely position firstly with a LOD score threshold cut off of LOD3 (the standard threshold for framework markers) which represents a 1:1000 chance of a marker being placed incorrectly. After placement of the LOD3 markers, the omitted non-framework markers and remaining secondary haplogroup markers were incorporated into the framework map using successive BUILD commands at the following decreasing LOD threshold cut offs; LOD2 (1:100 chance of incorrect marker placement), LOD1 (1:10 chance of incorrect marker placement) and finally the most likely position of remaining loci. For each BUILD at each LOD threshold cut-off, the marker order was verified using the FLIPS function with a moving window of five markers (FLIPS5). When a better marker order was established after FLIPS5, marker order was resolved and FLIPS5 was re-run until no further changes were apparent. CHROMPIC was then employed to ensure no incorrect double recombinants were present which may indicate incorrect marker positioning. Erroneous genotype calls were corrected and any markers identified with double recombinants were reanalysed with BUILD and FLIPS to determine if the double recombinants were real or the marker position was incorrect. Any markers with unresolved double recombinants were excluded and FLIPS5 was re-run to ensure the remaining marker order remained correct. The final map is referred to as the comprehensive map [23]. Sex-specific maps were also constructed using the sex-average marker order and recalculating marker intervals based on separate male and female informative meiosis events. Final map distances were calculated using the option FIXED. The Kosambi mapping function [24] was used for all cM calculations and all maps were drawn using MapDraw version 2.2 [25].

To validate the map ordering of CRI-MAP, markers belonging to a large linkage group with a range of informative loci (LG8 - established by AUTOGROUP in CRI-MAP) were chosen to build an independent sex-average comprehensive linkage map with CarthaGène version 1.0 which incorporates an EM (expectation-maximization) algorithm and a local search technique to build a maximum likelihood map [26]. The phasing function in TMAP version 1.1 [27] was utilised to generate input files for CarthaGène which incorporated the eight reference mapping families (Figure 1). The map was built using the same hierarchical mapping LOD thresholds as outlined above (LOD3, LOD2, LOD1 and most likely position) using recurrent executions of "build", "polish" and "flips".

To calculate genome coverage of the linkage maps the observed and expected genome lengths need to be established. The observed genome length (Goa) was simply the addition of all observed linkage group lengths and the expected genome length (Ge) was calculated by multiplying the length (cM) of each linkage group by (*m* + 1)/(*m* - 1), where *m* is the number of loci in each linkage group see [28]. The total expected genome length was the sum of Ge from all linkage groups. Genome coverage (Coa), was calculated by dividing Goa by Ge see [29].

### Segregation distortion

Segregation distortion, defined as the deviation from Mendelian inheritance of co-dominant alleles, may be present as a result of gametic selection or post-zygotic selection. To determine if such biological processes are present, segregation distortion was investigated using log-likelihood ratio tests for goodness of fit to Mendelian expectations in the software suit LINKMFEX version 2.4 [30]. Here, G-values were calculated for all markers across all mothers and fathers of each family and subsequently tested using the heterogeneity G-test as described in Sokal and Rohlf [31]. For each marker, G Total (sum of G values across all parents) and G-Pooled [calculated from the sum of allele specific (A and B) and total numbers (N) of co-informative events] were calculated and compared to determine the direction of the distortion if present. Heterogeneity was then calculated by subtracting the Total G value from the Pooled G value [31].

### Sex-specific and family-specific recombination heterogeneity

Recombination heterogeneity is the difference in recombination rates at various levels throughout the data including between sexes and families. Significant recombination heterogeneity at any level can affect the estimates of mapping distances and its extent should be investigated [32]. To investigate sex-specific heterogeneity throughout independent linkage groups, the following goodness of fit heterogeneity test was utilised with one degree of freedom as described in Ott [33];

where,  is the joint sex-specific recombination rate and  represents the recombination rate when equal male and female recombination fractions are assumed. For each test, a false discovery rate (FDR) correction was applied to correct for multiple comparisons and minimise false positives [34].

To detect any differences in sex-specific recombination rates, ratios of female-to-male map distances were calculated (*R* = *X*_*f*_/*X*_*m*_) for each interval and linkage group as well as over the entire map. In addition, standardised marker interval distances were calculated for each sex [standardised interval distance = 100 * (interval distance/total LG length)] and plotted against one another. For all linkage groups, distinct slopes were observed along the length of the linkage group. Breakpoints between the distinct slopes for each linkage group were assigned by visual inspection. Each slope was analysed using a simple linear regression of two continuous variables (female and male) as they represent biologically real differences. For regression analysis, data that produced each slope were grouped into three groups (left, middle and right) for all linkage groups except LG7 & LG9 where only two groups were produced (Additional file 2).

To ensure any observed sex-specific recombination was truly due to differences between the sexes, and not affected by variation in individuals F_1_ parents, family specific heterogeneity was investigated for each F_1_ parent independently. LINKMFEX version 2.4 [30] was used to calculate the recombination fraction, number of co-informative meiotic events (N) and the number of recombinations (r) for all mapped locus intervals for the maternal and paternal lines of each family separately. The Zmax score (LOD) was calculated for the mother and father in each family, and combined across all mothers and fathers respectively using methods outlined in Ott [33]. The following M-test was employed to investigate individual F_1_ recombination heterogeneity within each mapping family [33].

Here,  represents the LOD scores maximum likelihood estimation (MLE) for the *i*th F_1_ reference family for a pair of markers, with  being the total LOD score MLE of all *i*th reference families.

### Extent of linkage disequilibrium

The extent of LD is an important consideration for association mapping as it indicates the relative size of chromosomal segments shared amongst individuals within a population, and thus determines the number of theoretical markers necessary to detect genetic associations to quantitative traits [35]. Two commonly used estimates of LD, *r*^*2*^[36] and *D’*[37], were computed using GOLD software [38]. The LD estimates were computed among all 1,167 SNPs using genotypic data on 995 oysters (the additional 660 oysters either have no pedigree information or belong to smaller families not suitable for linkage mapping). The extent of LD among SNPs, within and across the linkage groups, was estimated using position of SNPs on the current linkage map.

## Results

### Genotyping, pedigrees and data integrity

The validation success of SNPs included on the custom genotyping array is detailed in Jones et al. [15]. Strict data integrity on the SNPs based on a genotyped population consisting of 525 individuals produced a total of 1,189 SNPs suitable for linkage mapping [15]. Of these, 1,167 produced polymorphic genotypes (MAF > 0.01) across the subset of 351 oysters belonging to the reference mapping families with an average genotyping call rate of > 99.4% (Additional file 1).

### Sex-average map

**Figure 2:**
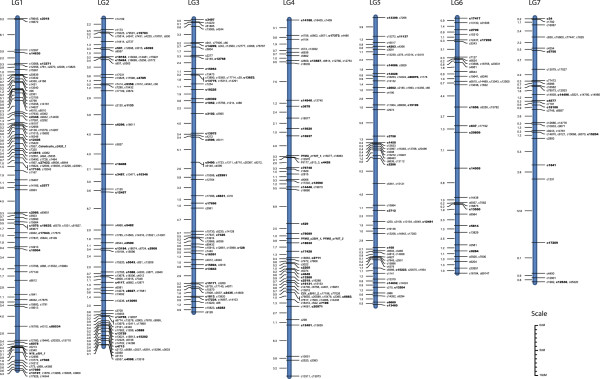
**The sex-average maps for linkage groups 1–7.** SNP IDs in bold indicate framework SNPs placed at a LOD > 3 and remaining SNPs have been placed in their most likely position at a LOD < 3. SNPs located within known biomineralization genes are indicated in bold italics.

**Figure 3:**
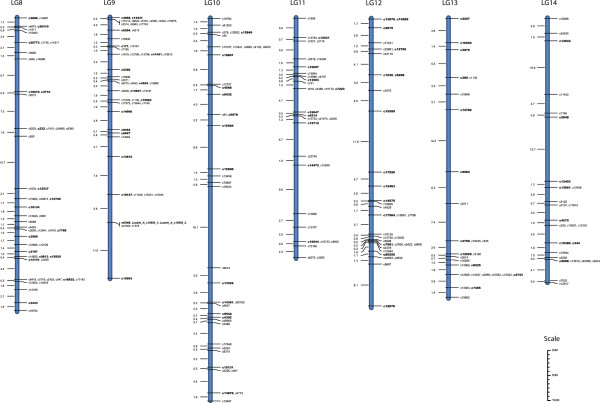
**The sex-average maps for linkage groups 8–14.** SNP IDs in bold indicate framework SNPs placed at a LOD > 3 and remaining SNPs have been placed in their most likely position at a LOD < 3. SNPs located within known biomineralization genes are indicated in bold italics.

**Figure 4:**
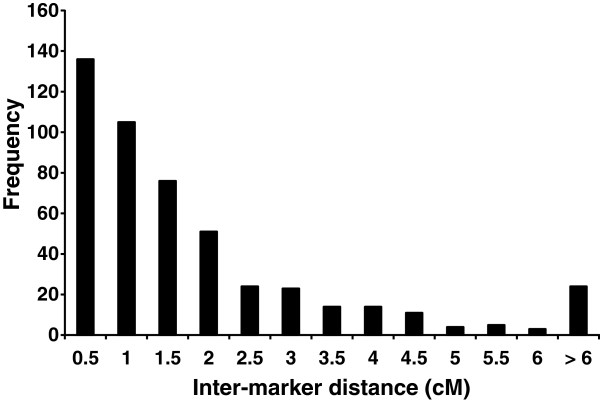
**Frequency of the sex-average inter-marker distances (cM) across the fourteen** ***P. maxima*** **linkage groups.** Only intervals > 0 cM were included. Over 49% of all intervals are below 1 cM, demonstrating an even spread of markers throughout the genome.

Genetic data used to construct our *P. maxima* linkage map consisted of 80,377 phase known and 259,844 phase unknown informative meiosis events across all 1,167 SNPs. The number of informative meiosis per marker ranged from 0 to 219 (average 68.17) for phase known, and 0 to 593 (average 220.39) for phase unknown. Of the 1,167 SNPs that passed quality criteria, 125 had less than ten informative meiosis events (either phase known or phase unknown) and were excluded from further analysis. A further 49 SNPs were not placed in linkage groups during AUTOGROUP. The remaining 993 SNPs were subsequently grouped into one of the 14 linkage groups. A total of 887 SNPs were successfully mapped to their most likely position within one of the 14 linkage groups with no ambiguity (Figures 2 and 3, and Additional files 1 and 3, 4, 5, 6, 7, 8, 9, 10, 11, 12, 13, 14, 15 and 16). The 106 grouped but unmapped SNPs could not be assigned a unique position as they exhibited low numbers of pairwise informative meiosis events (average phase known informative meiosis events 29.8) resulting in low power to resolve positions for these markers. This sex-average map spans 96.1% (831.7 cM) of the total estimated genome length (865.6 cM) (Table 1), with the average marker interval being 2.0 cM (when pairwise intervals of 0 cM were excluded). The two largest linkage groups (LGs), LG1 and LG2, both had 129 mapped markers, and spanned 70.3 cM and 66.3 cM respectively. LG13 and LG14 contained the fewest markers at 27 and 26 respectively and spanned 55.4 cM and 52.1 cM. Over 49% of the inter-marker distances were less than 1 cM and the median inter-marker genetic distance throughout the map (including inter-marker intervals of 0 cM) is 1.0 cM (range from 0.0 cM to 16.0 cM) (Figure 4). The map length of the *P. maxima* linkage groups ranged from 48.3 cM to 75.6 cM and exhibited a negative correlation with the number of markers mapped per linkage group (Table 1). Independent map ordering of LG8 using CarthaGène software confirmed the positions of all LOD3 (framework), LOD2 and LOD1 placed markers, indicating that generated maps are highly reproducible regardless of mapping algorithms and methods. Only four re-arrangements of markers placed in their most likely position were detected (c7736, c4016, c17142, c2359). For each of these rearrangements, the placement of the CarthaGène map was less than three positions away from the placement on the CRI-MAP map and the average distance between the alternative positions was 0.9 cM.

**Table 1:** **Summary statistics of the sex**-**average, female and male linkage maps of** ***P. maxima***

		Map length (cM)			Expected genome length (Ge)				Average interval (cM)			No of intervals (Sex-Av.)				
LG	No. of SNPs	Sex-Av.	Female	Male	Sex-Av.	Female	Male	Female: Male ratio	Sex-Av. (SD)	Female (SD)	Male (SD)	All	0-1 cM	1-2 cM	2-3 cM	>3 cM
1	129	70.30	65.53	75.29	71.40	66.55	76.46	0.87	0.99 (+/- 1.09)	1.34 (+/- 1.90)	1.30 (+/- 1.25)	71	49	14	4	4
2	129	66.27	65.12	66.02	67.31	66.14	67.05	0.99	1.20 (+/- 1.29)	1.63 (+/- 2.82)	1.18 (+/- 1.21)	55	33	11	6	5
3	97	59.36	63.96	55.25	60.60	65.29	56.41	1.16	1.14 (+/- 1.21)	2.00 (+/- 2.72)	1.67 (+/- 1.49)	52	31	12	4	5
4	89	71.86	76.94	83.76	73.49	78.69	85.66	0.92	1.41 (+/- 1.64)	2.56 (+/- 3.04)	2.54 (+/- 3.16)	51	27	14	4	6
5	82	60.02	66.78	61.63	61.50	68.43	63.15	1.08	1.28 (+/- 1.27)	1.96 (+/- 3.54)	1.81 (+/- 1.72)	47	24	15	4	4
6	46	50.35	57.52	47.35	52.59	60.07	49.46	1.21	1.68 (+/- 1.64)	3.20 (+/- 3.99)	1.58 (+/- 1.51)	30	12	11	2	5
7	46	52.33	68.90	45.13	54.65	71.96	47.13	1.53	1.94 (+/- 2.62)	6.26 (+/- 9.67)	1.88 (+/- 2.16)	27	13	5	5	4
8	55	59.98	68.53	49.12	62.20	71.07	50.94	1.40	2.14 (+/- 2.37)	4.03 (+/- 6.76)	1.64 (+/- 1.70)	28	8	12	4	4
9	53	54.18	43.18	58.91	56.26	44.84	61.17	0.73	2.36 (+/- 2.80)	5.40 (+/- 7.15)	2.81 (+/- 1.70)	23	9	7	1	6
10	40	75.56	83.88	74.40	79.43	88.18	78.21	1.13	2.61 (+/- 3.23)	4.41 (+/- 5.68)	2.57 (+/- 3.00)	29	10	8	2	9
11	34	48.29	59.08	33.20	51.21	62.66	35.21	1.78	2.30 (+/- 2.42)	3.94 (+/- 4.32)	2.08 (+/- 1.71)	21	8	5	3	5
12	34	55.68	58.78	52.49	59.05	62.34	55.67	1.12	2.42 (+/- 2.75)	3.46 (+/- 3.39)	3.28 (+/- 3.75)	23	8	6	4	5
13	27	55.36	77.13	36.34	59.62	83.06	39.14	2.12	3.46 (+/- 3.18)	6.43 (+/- 7.07)	2.60 (+/- 2.36)	16	3	4	2	7
14	26	52.13	60.52	55.96	56.30	65.36	60.44	1.08	3.07 (+/- 3.48)	5.04 (+/- 8.09)	3.50 (+/- 1.96)	17	6	3	2	6
**Total**	**887**	**831.66**	**915.83**	**794.84**	**865.62**	**954.64**	**826.10**	**1.15**	**2.00**	**3.69**	**2.17**	**490**	**241**	**127**	**47**	**75**
**Genome coverage**		**96.08%**	**95.93%**	**96.22%**												

Observed map length (cM), expected genome length (Ge) and average intervals are reported for the sex-average (Sex Av.), female and male maps of *Pinctada maxima*. In addition, the female-to-male recombination ratios and number of intervals for the sex-average map are included.

### Sex-specific and family-specific recombination heterogeneity

**Figure 5:**
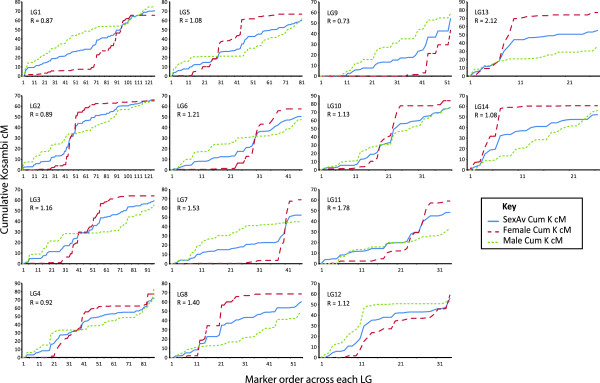
**The cumulative Kosambi cM for the sex-average, female and male maps.** The extent and patterns of localised regional sex-specific recombination rates are illustrated for each linkage group. The overall female-to-male ratio (R) for each linkage group is also reported.

**Figure 6:**
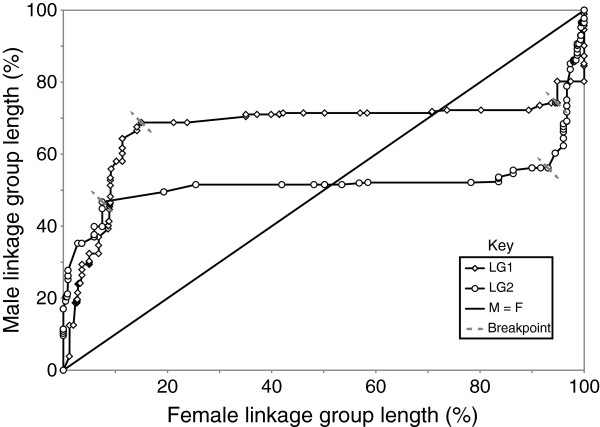
**Comparison of standardised female and male interval distances of LG1 and LG2 revealing highly variable sex-specific recombination along both linkage groups.** Regression analysis was performed by visually determining breakpoints (dashed lines) and grouping data into three slopes, left, middle and right. The male map is compressed near the centromeres and expanded near the telomeres, and the opposite was observed for the female map. The average slope of the lines in the two middle sections (centromeric) is 0.07 (±0.02) and is significantly different from 1 (P < 0.05). The average male-to-female recombination ratio for the slopes near the centromere is 1:5.98, indicating a male "cold-spot" for recombination. The average slope of the lines near the telomeres are 4.29 (±0.56) for the left group and 5.20 (±3.06) for the right, and again are significantly different from 1 (P < 0.05).

Sex-specific maps were produced using the sex-average marker order to recalculate marker intervals based on 37,306 phase known and 130,179 phase unknown meiotic events for the male map, and 43,071 phase known and 129,665 phase unknown meiotic events for the female map. Significant differences in sex-specific recombination were observed for all linkage groups and the entire map (Heterogeneity Test *P* values < 0.001, Figures 5 and 6, and Additional files 3, 4, 5, 6, 7, 8, 9, 10, 11, 12, 13, 14, 15 and 16). Out of the 14 linkage groups, 10 (LG3, LG5-8 and LG10-14) displayed slightly larger female maps relative to male maps. Overall the observed female sex-specific map was 121.0 cM larger than the observed male map, with an average female-to-male ratio of 1.15:1 (Table 1). The sex-specific log likelihood for each linkage group, averaged between the sexes, ranged from -346.1 to -759.0 (average -536.886) and the total sex-specific log likelihood was -7516.4.

**Figure 7:**
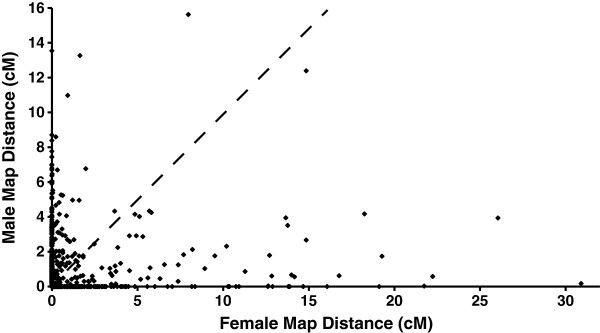
**A plot of the female vs male inter-marker distances (cM) for all pairs of adjacent markers.** The dashed line represents a 1:1 sex ratio whereby recombination is the same in both sexes. The majority of the points fall close to either 0 on the x-axis, or 0 on the y-axis indicating both strong female biased and strong male biased recombination throughout all intervals.

Female-to-male ratios (F:M ratios) of inter-marker distances deviated substantially from the expected 1:1 ratio and were either close to zero or very large indicating pronounced localised differences in recombination rates between the sexes (Figure 7). Distinct patterns of sex-specific recombination throughout the linkage groups were observed, whereby recombination rates were usually greater towards the end of the linkage groups and suppressed in centromeric positions for the male map, with the opposite pattern being observed for the female map (Figures 5 and 6). As a result, clustering of markers was observed towards the centre of the linkage groups in the male map and at the end of the linkage groups in the female map (Additional files 3, 4, 5, 6, 7, 8, 9, 10, 11, 12, 13, 14, 15 and 16). Mild to strong localised sex-specific recombination patterns were prevalent over 11 linkage groups (LG1-LG8, LG10-LG11 & LG13) as illustrated by plots of the sex-average, female and male cumulative cM throughout each linkage group (Figure 5) and the regression analysis of standardised sex-specific interval sizes (Figure 6 and Additional files 2, 17, 18 and 19). In addition, comparisons of standardised interval sizes for female and male maps along LG1 and LG2 also confirm this pattern (Figure 6). After dividing the standardised interval distances for LG1 and LG2 into groups (based on breakpoints) for regression analysis, the mean slope of the two lines in the middle group of the graph (centromeric) is 0.1 (± 0.02), and is significantly less than 1 (*P* < 0.05), the slope expected if there was no difference in the sex-specific recombination rates. This indicates that most of the reduction in male recombination rates is taking place in the centre of the linkage groups. In contrast, the average slope near the telomeres of the linkage groups for the left and right groups were 4.3 (±0.6) and 5.2 (± 3.1), respectively, and significantly greater than 1 (*P* < 0.05). Based on this, male recombination rates are larger relative to female rates in telemetric regions.

Investigations into family specific heterogeneity confirm that observed sex-specific recombination is truly caused by the sexes and not individual parental F_1_ individuals biasing the data. Only one interval in LG1 on the sex-average map (c10004 - c13798) returned significant recombination heterogeneity after FDR (*χ*^*2*^ = 21.6, *P* = 0.0002, df = 4). This deviation was explained by deviations in only two of the eight families (130×148 and 131×145) providing evidence that the mapping parents are relatively homogeneous within the sexes for recombination differences.

### Segregation distortions

Significant segregation distortions were detected in 121 of the 887 mapped SNPs and seven of the sixteen mapping parents following FDR correction (mean corrected alpha of 0.003) (Additional file 20). The majority (64.9%) of these distortions were localized to the mapping family 103×102 and to linkage groups 2, 4 and 10. As no significant family specific heterogeneity was detected for these distortions, they are not thought to be influencing calculations of mapping distances. However, to be conservative, only markers that did not cause conflicts in map position were mapped.

### Biomineralization gene mapping

Positional information of biomineralization candidate genes can assist in determining which genes influence pearl quality traits by comparing their positions to QTLs. As described in Jones et al. [15], numerous SNPs were designed within known biomineralization genes. A total of nine SNPs designed within six biomineralization gene homologs were successfully mapped. These genes were Calreticulin, chitin synthase 1 (CS1), Lustrin A, N19, *Pinctada fucata* mantle gene (PFMG) complex and Pif177. Two SNPs from Lustin A were mapped, clustering together in a telomeric region of LG9 (Figure 3) and three SNPs designed within the PFMG complex were mapped to the centre of LG4 (Figure 2) along with the SNPs designed in Pif177 and CS1. A SNP from Calreticulin was mapped to the centre of LG1 and N19 was mapped to the end of LG1 (Figure 2).

### Extent of linkage disequilibrium

**Figure 8:**
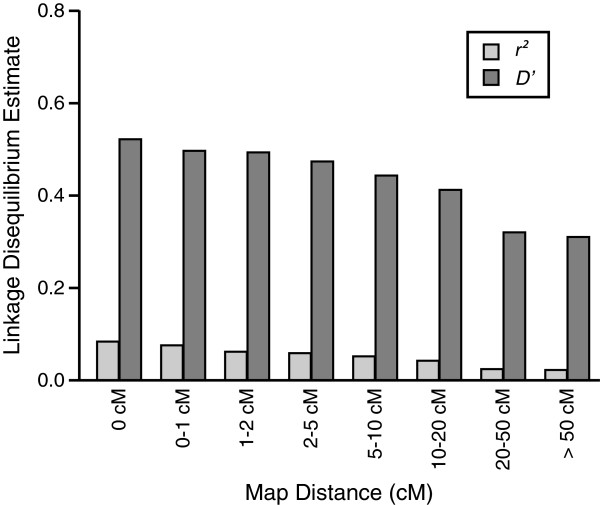
**Mean linkage disequilibrium (LD) estimates at different linkage map distances throughout the** ***P. maxima*** **genome for** ***r*** ^***2***^ **and D’.**

Overall distributions of LD estimates for syntenic (on the same linkage group) and non-syntenic (on different linkage groups) SNP pairs (Table 2) indicate a larger proportion of non-syntenic pairs have small values of LD estimates (< 0.1). The mean (first and third quartile) of *r*^*2*^ for 357,025 non-syntenic pairs is 0.014 (0.001, 0.019) and *D’* is 0.263 (0.079 and 0.362). As expected, these non-syntenic LD estimates are slightly lower as compared to among syntenic SNPs located more than 50 cM apart, where mean *r*^*2*^ and *D’* estimated were 0.02 and 0.31 respectively. LD estimates declined gradually over increasing map distances throughout the genome (Table 3 and Figure 8). Variation in the trends of decline in LD estimates for individual linkage groups are presented in Additional file 21. For example, LG10-12 show a steeper trend of decline of LD estimates over increasing map distances.

**Table 2:** **Overall distribution of linkage disequilibrium (LD) estimates (** ***r*** ^***2***^ **and** ***D’*** **) for all, non-syntenic and syntenic SNPs**

Range of estimate	Number of SNP pairs					
	All		Non-syntenic		Syntenic	
	***r*** ^***2***^	***D’***	***r*** ^***2***^	***D’***	***r*** ^***2***^	***D’***
**0**	100527	1287	52982	845	3459	47
**0 - 0.1**	546940	172025	300505	110217	29079	6844
**0.1 - 0.2**	8115	130826	3345	81149	2330	5846
**0.2 - 0.3**	1091	90856	178	53175	622	4698
**0.3 - 0.4**	332	62526	14	33430	244	3976
**0.4 - 0.5**	117	44618	0	22083	98	3231
**0.5 - 0.6**	50	33147	1	14986	36	2566
**0.6 - 0.7**	27	26074	0	10869	23	2302
**0.7 - 0.8**	10	21640	0	8427	7	1864
**0.8 - 0.9**	4	18568	0	6898	4	1639
**0.9 - 1**	18	55664	0	14946	14	2903

Estimate of LD for all SNPs are based on all 1,167 available SNPs; estimates of LD for non-syntenic SNPs are based on mapped SNP pairs located on different linkage groups; and estimates of LD for syntenic SNPs are based on mapped SNP pairs located on the same linkage group.

**Table 3:** **Mean (± SD) and median of** ***r*** ^***2***^ **and** ***D’*** **linkage disequilibrium estimates over distance for all linkage groups**

Distance	***N***	***r*** ^***2***^mean (± SD)	***r*** ^***2***^median	***D’*** mean (± SD)	***D’*** median
**0 cM**	740	0.082 (± 0.138)	0.032	0.519 (± 0.321)	0.489
**0 - 1 cM**	991	0.075 (± 0.125)	0.031	0.494 (± 0.323)	0.477
**1 - 2 cM**	1254	0.061 (± 0.094)	0.025	0.490 (± 0.311)	0.465
**2 - 5 cM**	3440	0.058 (± 0.088)	0.024	0.472 (± 0.315)	0.438
**5 - 10 cM**	4997	0.051 (± 0.078)	0.022	0.440 (± 0.299)	0.396
**10 - 20 cM**	7079	0.042 (± 0.064)	0.018	0.410 (± 0.293)	0.360
**20 - 50 cM**	14087	0.022 (± 0.034)	0.011	0.319 (± 0.265)	0.245
**> 50 cM**	3328	0.020 (± 0.029)	0.009	0.307 (± 0.266)	0.230

## Discussion

The robust high-density genetic linkage map for *P. maxima* presented here is the most comprehensive map to date for any bivalve species. With the combination of physical mapping data, this map will contribute to a better understanding of the genome structure, function and evolution for *P. maxima*, and other species within the genus *Pinctada*. In addition, the identification of genetic associations and QTLs for commercial traits will be highly valuable to the pearling industry as this information will be directly used for genetic improvement of complex traits in farmed stock.

### Genotyping, marker selection and pedigree validation

Missing genotypes or typing errors are known to interfere with the ordering of SNPs leading to incorrect estimation of map lengths [20]. Therefore, thorough genotypic data integrity is vital for the generation of accurate maps. The majority of data integrity applied to this dataset has been described in Jones et al. [15]. This previous work has provided a highly refined list of SNPs useful for linkage mapping analysis by excluding all SNPs with low polymorphism and removing erroneous genotype errors including deviations from HWE, Mendelian incompatibility, SNP duplication, low MAF and low call rates. The average number of informative meiosis events (83,377 phase known and 259,844 phase unknown for sex-average) for this subset of 1,167 SNPs over our eight families was high ensuring fine resolution throughout the map. However, in some cases, the order of closely linked markers (N = 397, recombination = 0) could not be determined even though the overall number of informative meiosis was relatively high. For these loci, a small proportion cannot be separated due to limitations in SNP discovery see [15], while others will require more informative meiosis events to eventually separate.

Parental relationships of mapping families utilised in this study were rigorously tested and confirmed using parentage analysis during previous projects, minimising the possibility of pedigree errors. This was further confirmed through testing for MI errors during map construction. No families were found with Mendelian Inheritance errors across many informative loci. Overall, the level of Mendelian inconsistencies was extremely low for a custom array consisting of novel SNPs. A few sporadic MI errors could be attributed to poor DNA quality in a few samples which were subsequently removed.

### Segregation distortion

One of the problems in linkage mapping of oysters is that moderate distortions from expected Mendelian segregation are common [5]. Markers that exhibit segregation distortion can potentially influence marker positions and linkage relationships, however, the presence of moderate segregation distortion has been reported to have little effect on the overall construction of linkage maps [20, 39], and maps have been successfully constructed in species exhibiting moderate segregation distortions [40]. Additionally, the inclusion of distorted markers in mapping can be beneficial as they may have an association to genes that affect fitness and survival, particularly larval mortality, and they may also help with understanding the distribution of deleterious recessive genes throughout the genome.

The extent of segregation distortion throughout the *P. maxima* linkage map was investigated to determine their influence on marker order and mapping distances and to determine if markers exhibiting distortion clustered together. A total of 121 mapped SNP loci showed at least one significant distortion in a family after stringent FDR correction (average FDR alpha value of 0.0032). A high proportion of these distortions (79.8%) were localised to specific linkage groups (25.4% to LG1, 20.9% to LG2, 15.7% to LG4, 9.7% to LG3 and 8.2% to LG10) indicating a true biological phenomenon is in effect instead of random artefacts [20]. Localised segregation distortion has been reported as a common feature in numerous bivalve species including, the Pacific oyster (*Crassostrea gigas*) [9, 39, 41], the European flat oyster (*Ostrea edulis*) [11] and *Pinctada martensii*[12]. Since at least *C. gigas* is known to have a high genetic load [42], such distortions in these bivalves have been explained by zygotic viability selection due to the presence of deleterious recessive genes [9, 11, 12]. Segregation distortions reported here may also be attributed to the presence of deleterious recessive genes as has been observed in *C. gigas*, *O. edulis* and *P. martensii*, however, further research is warranted to confirm this.

### Map construction and estimated genome size

Linkage map construction resulted in the generation of 14 linkage groups that correspond to the 14 haploid chromosomes of *P. maxima*[7]. Approximately 76% of the SNPs (887 out of 1,167) were placed on the linkage map (Additional file 1). This comprehensive first-generation linkage map is a substantial resource and is a large improvement on any bivalve map to date with reference to the number of markers mapped (previous average number of markers mapped of 191) and genome coverage (previous average genome coverage of 80.4%) [8–12, 43]. The number of markers on this map (*N* = 887) more than doubles any previous attempt in bivalves and the predicted genome coverage (96%) is much higher than an average of 80% reported in previous bivalve maps. In addition, the distribution of inter-SNP spacing throughout the map demonstrates an even spread of markers throughout the genome with over 49% of the inter-marker distances being less than 1 cM (Median inter-marker distance of 1.03 cM).

The estimated genome size for *P. maxima* based on the sex-average linkage map is 865.6 cM (954.6 cM for the female map and 826.1 cM for the male map). This is significantly less than expected genome length reported for a previous linkage map for *Pinctada martensii* (1862.9 cM for the female map and 1838.4 for the male map) [12]. Assuming similar recombination rates between the species, one possible explanation for this is that the inclusion of more markers refines positions and reduces the total cM of each linkage group as acknowledged by Shi et al. [12]. Maps of low density are commonly longer than maps of high density and as more markers are added, map length decreases [8, 9, 44]. A low marker density in the previous *P. martensii* map is most likely the cause of the overestimation of genome size. The length of the *P. maxima* linkage groups in present study varied from 48.3 cM to 75.6 cM and exhibited a negative relationship with the number of markers mapped per linkage group (Table 1). As a result, linkage groups of smaller sizes showed similar recombination rates than those of larger sizes. This too may be a result of inflated map distances for linkage groups with fewer markers mapped.

### Sex-specific maps and recombination rates

Sex-specific differences in recombination rates are not uncommon and have been reported in numerous vertebrate [45–48] and invertebrate [10, 12, 49, 50] species. In accordance to the Haldane rule, for organisms with a chromosomal mechanism of sex determination, recombination should be more frequent in the homogametic sex than in the heterogametic sex [32, 33, 51]. This observation has been termed heterochiasmy. However, many exceptions to this rule have been demonstrated including the tammar wallaby [45], the great reed warbler [46] and the saltwater crocodile [47]. In addition, reports of sex-specific recombination in species without heteromorphic sex chromosomes have become apparent [47, 48, 52, 53]. Oysters are one taxa that lack specialised heteromorphic sex chromosomes [7, 54], but exhibit sex-specific recombination [this study, 8, 9]. Results observed here for *P. maxima* show that the male map (826.1 cM) is shorter than the female map (954.64 cM), suggesting a slight female bias in recombination with an overall ratio of female-to-male recombination of 1.15:1 and ratios reaching 2.12:1 in LG13. This is comparable to previous ratios of sex-specific recombination in oysters that range from 1.07:1–1.51:1 [8, 9, 12] and other aquaculture species (female-to-male ratios range from 1.2:1 – 3.25:1) [48, 52, 55, 56]. Such proliferation of studies that report female biased sex-specific recombination in species with no specialised sex chromosomes suggest that there must be another underling phenomenon of the timing, duration or biological features associated with meiosis that is responsible for the observed differences between the sexes.

Sex-specific recombination rates are also known to differ throughout regions within the genome [57]. Dramatic localised sex-specific differences were detected throughout the *P. maxima* maps where male recombination rates were supressed relative to the female rates in areas proximal to centromeres, but elevated in regions distal to centromeres, with females showing the opposite pattern (see Figure 5 and Additional files 3, 4, 5, 6, 7, 8, 9, 10, 11, 12, 13, 14, 15 and 16) [58, 59]. The expansion of the male genetic map in telemetric regions indicates that chiasmata would be found more frequently near the telomeres in meiosis in males compared to females. Similarly, chiasmata would be more common in centromeric regions during oogenesis. Such pronounced localised differences in recombination rates have not been previously reported in oysters [10], but are quite widespread throughout other aquaculture species including the rainbow trout (*Oncorhynchus mykiss*) [55], Atlantic salmon (*Salmo salar*) [48] and the zebrafish (*Danio rerio*) [52]. This unusual pattern of sex-specific recombination is not well understood, however, several theories have been suggested [reviewed by Miles et al. [47]. Briefly, sex-specific recombination could have been caused by a) differing environments in which the germ cells develop [60], b) temporal differences in initiation of meiosis between the sexes [61] and c) differences in the pairing and synapses of homologs at meiosis that cause different exchange patterns in oocytes and spermatocytes [62]. However, further research is required to confirm these theories in *P. maxima*.

Nevertheless, it is remarkable that such strong sex-specific recombination patterns are present in a species without differentiated sex chromosomes, exhibits no sexual dimorphism and is a protandrous hermaphrodite (maturing first as a male and able to switch sex after 2 years of age). Definitely, the strong evidence of sex-specific recombination presented here may aid in identifying the mechanism behind sex-specific recombination, especially for species without differentiated sex chromosomes. To truly, elucidate the basis of sex-specific recombination, cytogenetic analysis of female and male meiosis would be required. The unusual life history of *P. maxima* (a protandrous hermaphrodite) may allow the estimation of female and male recombination rates in the same individual removing any effect of genetic background on such estimations. As suggested by Franch et al. [56], hermaphroditic species might play a crucial role in dissecting the contribution of sex-determining and sex-differentiating genes on meiotic recombination [56].

### Biomineralization gene mapping

Nine mapped SNPs were designed within six homolog sequences of known candidate genes for biomineralization (Calreticulin, CS1, Lustrin A, N19, PFMG complex and Pif177). Clustering of these SNPs in four locations within the genome (telemetric region of LG9, centre of LG4, centre of LG1 and telemetric region of LG1) provides evidence that these regions may have a strong influence on biomineralization for *P. maxima* and signposts these regions for further investigation to determine true associations to biomineralization processes. Five of the nine biomineralization gene SNPs clustered within 27.2 cM near the centre of LG4 (total length of 71.9 cM). These SNPs represented three genes, the PFMG1, Pif177 and CS1 which are highly expressed in mantle tissue of pearl oysters and are known to be involved in nacre formation [63–65]. More specifically, PFMG1 and Pif177 (both initially described in *P. fucata*) are key calcium-binding proteins that specifically bind aragonite crystals and regulate nucleation and precipitation during nacre formation [64, 65]. PFMG1 and Pif177 have also previously been co-localised during EST clustering analysis for *P. fucata* sequences [66]. The second gene, Pif177 consists of two proteins, Pif80 and Pif97 which are encoded by a single mRNA [65]. Pif80, Pif97 and N16 (another nacre biomineralization protein) work in collaboration (along with chitin) to initiate aragonite crystallization and orientate the stacking of aragonite tablets in nacreous layers [65, 67]. Interestingly, the third protein clustering at this region, CS1, is also involved in the gene complex described above. CS1 is a key enzyme responsible for the deposition of chitin, a polysaccharide integral for calcium carbonate biomineral formation in mollusc shells [63]. The co-localisation of these three major nacre biomineralization genes to central parts of LG4 provides strong evidence that this region is a hot spot for nacre biomineralization genes and would become a prime target for studies aiming to identify QTL for commercially valuable pearl quality traits.

Four additional SNPs designed within three genes (Lustrin A, Calreticulin and N19) were localised to three other regions throughout the linkage map. Two SNPs (Lustrin_A_c15856_1 and Lustrin_A_c15856_2) designed within a contig homologous to Lustrin A (c15856) were mapped to the same position (0 cM intermarker distance) close to a telomere of LG9. The SNP Calreticulin_c2420_1 (designed within a contig homologous to Calreticulin) was mapped to the centre of LG1 and N19_c591_1 (designed within a contig homologous to N19) was mapped to a telemetric region of LG1. Specific functions have been ascribed to each of these three genes, including; conferring elastic resilience to the molluscan shell and maintaining the structure and protein compounds of nacre for Lustrin A [68]; calcium binding, transport and storage during biomineralization for Calreticulin [69]; and finally, having a negative regulatory role in calcification for N19 [70]. The localisation of these six biomineralization genes will increase the accuracy of identifying regions of interest for researchers interested in identifying genetic association to important nacre biomineralization genes and will also be important for comparative mapping studies investigating genome evolution and synteny.

### Extent of linkage disequilibrium

Association studies aiming to identify genetic variations or quantitative trait nucleotide (QTN) that explain a large proportion of the phenotypic variance in a quantitative trait rely on the co-segregation of QTNs with the surrounding genetic markers or loci. If the marker and QTN are sufficiently close, this association will remain intact within the population over many generations [71]. Such non-random association between loci is termed linkage disequilibrium (LD). The extent of LD is therefore important as it defines the density of genome-wide makers necessary for association analysis to detect markers associated with traits of commercial interest and are also in LD with QTNs. Generally, higher marker density is beneficial, although, if the extent of LD throughput the genome is high, fewer markers may be sufficient for association studies [35]. Two estimates of the extent of LD were utilised in this study, *D’* and *r*^*2*^. The *D’* estimate of LD is suggested to be a good measure for the extent of LD in a population and variation in LD throughout the genome as it focuses on historical recombination. However, *D’* is known to be more influenced by allelic frequency variation than the *r*^*2*^ estimate [35]. As such, *r*^*2*^ is more useful in predicting the power of association mapping.

The LD estimates presented in this study are based on 995 oysters which include an additional 660 oysters to those utilised for linkage mapping. Additional animals from smaller families and unknown pedigree are particularly suitable for computing LD estimates. Estimates of LD among non-syntenic (on different chromosomes) SNP pairs represent background variation observed within the data. The mean estimate of LD among non-syntenic SNPs for *P. maxima* (mean *r*^*2*^ of 0.020) is generally higher when compared to well characterised species (i.e. bovine with mean *r*^*2*^ of 0.003; [35]). This may be due to the high relationship among animals in this population as compared to bovine. Therefore, the comparatively higher background LD estimates of non-syntenic SNPs in this study are not unexpected. For syntenic (on the same chromosome) SNP pairs greater than 50 cM apart, estimates of LD were similar to that of non-syntenic SNPs (*r*^*2*^ and *D’* of 0.014 and 0.307 respectively). This indicates that recombination between these long range SNP pairs is relatively high, and SNPs on distal ends of the chromosomes are behaving in a similar manner as non-syntenic SNPs.

Estimates of LD usually decline as map distance increases in most species. Here, the decline in LD over map distance is gradual for both *D’* and *r*^*2*^ estimates in *P. maxima* (Table 3 and Figure 8). However, the mean LD estimates among closely spaced markers are lower as compared to other well characterised species (e.g. bovine, human [35]) which suggests a low extent of LD within the current population. Limited studies of LD have been reported in invertebrates. The LD estimates reported here are contrary to what has been observed in another aquaculture species, the Pacific white shrimp (*Litopenaeus vannamei*). For *L. vannamei*, a steeper decline in LD with map distance suggests smaller LD blocks [72]. In addition, estimates of *r*^*2*^ for syntenic SNP pairs greater than 50 cM apart were higher than that observed in this study (0.15 compared to 0.014). This is likely due to a difference in the effective population size (*N*_*e*_) between the two studies. The current study was based on multiple families derived from outbred populations (higher *N*_*e*_), whereas, estimates of LD for *L. vannamei* were based on only 144 individuals from six family lines (lower *N*_*e*_). However, the LD estimates of *L. vannamei* are probably more typical of aquaculture species in general as these are usually derived from limited numbers of stocks.

The low LD estimates for short range (0–1 cM) syntenic SNPs (*r*^*2*^ and *D’* of 0.083 and 0.519 respectively), and gradual decline in LD, suggests limited short range LD at the current marker density. To fully evaluate short range LD in this population, marker density needs to be increased. As a result, these LD estimates must be treated with caution. With a higher marker density, the decline of LD throughout the genome may be more pronounced once better estimates can be made between SNPs pairs at smaller map intervals. Furthermore, in this study, the extent of LD was compared against the linkage map, however, both linkage and LD maps are calculated using recombination rates. The extent of LD across a genome is better understood when presented against the physical map positions. Nevertheless, in the absence of a physical map, these results provide a preliminary estimate of broad patterns of LD observed within the oyster genome for this population. Even though the present SNP density will be useful for first-pass QTL and genome-wide association studies (GWAS), the low values of *r*^*2*^ for most adjacent SNPs pairs suggest that density should be increased before fine-scale trait and LD mapping across the *P. maxima* genome is attempted.

## Conclusions

This research developed a high-density genetic linkage map suitable for studies aiming to identify gene associations and QTLs for commercially important traits such as shell growth, pearl size, nacre colour and surface complexion in the silver-lipped pearl oyster. The genetic linkage map will be particularly useful for the mapping of QTLs in this species, especially since it is of high density, the mapped SNPs are genic, and numerous regions have been flagged with genes known to be involved in nacre biomineralization. The density of this linkage map would also be sufficient for preliminary GWAS analysis, however, higher density would be more appropriate considering the low extent of LD throughout the genome.

Finally, the transferability of mapped SNPs to species within the genus *Pinctada* has previously been shown to be high [15]. For example, conversion rates of SNPs between species closely related to *P. maxima* (i.e. 61.3% in *P. margaritifera* and 58.5% in *P. mazatlantica*), illustrate the high utility for the *P. maxima* map in comparative mapping studies. When other genomic resources become available for bivalve species, comparative mapping studies utilising our linkage map will provide insights into many fundamental questions in the localization of genes, conservation of gene content and order, genome evolution and synteny in bivalves.

## Electronic supplementary material

Additional file 1: **Detailed statistics for all 1167 SNPs deemed suitable for mapping analysis.** Detailed statistics on all 1167 SNPs suitable for mapping analysis. The source sequence from which the SNPs were designed is reported along with minor allele frequency and sequence length. All SNPs were assigned a destination of either 'Uninformative’: not returning sufficient informative meiosis within the mapping families mapped; 'Not assigned to LG’: returned informative meiosis but was not included in a LG; 'Assigned to LG but not mapped’: SNP was clustered during initial mapping but could not be positioned unambiguously; or finally 'Mapped’: SNPs which appear in the final comprehensive map. Map linkage groups, positions, informative meiosis and LOD placement cut-off are listed as well as the Kosambi cM for the sex-average, female and male maps. (XLSX 425 KB)

Additional file 2: **Regression statistics for all data groups across all linkage groups.** ANOVA that test the difference between the standardised female and male interval distances are also included. (XLSX 18 KB)

Additional file 3: **The sex-average and sex-specific (female and male) maps for linkage group 1.** SNP IDs in bold indicate framework SNPs placed at a LOD > 3 and remaining SNPs have been placed in their most likely position at a LOD < 3. SNPs located within known biomineralization genes are indicated in bold italics. Dotted lines indicate the respective placements of a few framework SNPs on the female and male maps. For LG1, the sex-average log likelihood is -834.2, the sex-specific (f,m) is -759.0, and the *P* value of the sex-specific heterogeneity test is highly significant at 2.8E-77 (FDR alpha value of 0.004). (EPS 2 MB)

Additional file 4: **The sex-average and sex-specific (female and male) maps for linkage group 2.** For LG2, the sex-average log likelihood is -781.2, the sex-specific (f,m) is -717.7, and the *P* value of the sex-specific heterogeneity test is highly significant at 1.5E-65 (FDR alpha value of 0.004). (EPS 2 MB)

Additional file 5: **The sex-average and sex-specific (female and male) maps for linkage group 3.** For LG3, the sex-average log likelihood is -747.6, the sex-specific (f,m) is -669.2, and the *P* value of the sex-specific heterogeneity test is highly significant at 1.9E-80 (FDR alpha value of 0.004). (EPS 1 MB)

Additional file 6: **The sex-average and sex-specific (female and male) maps for linkage group 4.** For LG4, the sex-average log likelihood is -762.1, the sex-specific (f,m) is -685.6, and the *P* value of the sex-specific heterogeneity test is highly significant at 1.3E-78 (FDR alpha value of 0.004). (EPS 2 MB)

Additional file 7: **The sex-average and sex-specific (female and male) maps for linkage group 5.** For LG5, the sex-average log likelihood is -680.5, the sex-specific (f,m) is -601.9, and the *P* value of the sex-specific heterogeneity test is highly significant at 9.2E-81 (FDR alpha value of 0.004). (EPS 1 MB)

Additional file 8: **The sex-average and sex-specific (female and male) maps for linkage group 6.** For LG6, the sex-average log likelihood is -552.9, the sex-specific (f,m) is -495.0, and the *P* value of the sex-specific heterogeneity test is highly significant at 5.8E-60 (FDR alpha value of 0.004). (EPS 1 MB)

Additional file 9: **The sex-average and sex-specific (female and male) maps for linkage group 7.** For LG7, the sex-average log likelihood is -495.7, the sex-specific (f,m) is -418.0, and the *P* value of the sex-specific heterogeneity test is highly significant at 8.3E-80 (FDR alpha value of 0.004). (EPS 1 MB)

Additional file 10: **The sex-average and sex-specific (female and male) maps for linkage group 8.** For LG8, the sex-average log likelihood is -600.5, the sex-specific (f,m) is -539.2, and the *P* value of the sex-specific heterogeneity test is highly significant at 2.3E-63 (FDR alpha value of 0.004). (EPS 1 MB)

Additional file 11: **The sex-average and sex-specific (female and male) maps for linkage group 9.** For LG9, the sex-average log likelihood is -489.4, the sex-specific (f,m) is -442.9, and the *P* value of the sex-specific heterogeneity test is highly significant at 1.6E-48 (FDR alpha value of 0.004). (EPS 1 MB)

Additional file 12: **The sex-average and sex-specific (female and male) maps for linkage group 10.** For LG10, the sex-average log likelihood is -627.4, the sex-specific (f,m) is -584.6, and the *P* value of the sex-specific heterogeneity test is highly significant at 9.8E-45 (FDR alpha value of 0.004). (EPS 1 MB)

Additional file 13: **The sex-average and sex-specific (female and male) maps for linkage group 11.** For LG11, the sex-average log likelihood is -430.2, the sex-specific (f,m) is -400.2, and the *P* value of the sex-specific heterogeneity test is highly significant at 7.8E-32 (FDR alpha value of 0.004). (EPS 1 MB)

Additional file 14: **The sex-average and sex-specific (female and male) maps for linkage group 12.** For LG12, the sex-average log likelihood is -518.4, the sex-specific (f,m) is -481.0, and the *P* value of the sex-specific heterogeneity test is highly significant at 2.4E-39 (FDR alpha value of 0.004). (EPS 1 MB)

Additional file 15: **The sex-average and sex-specific (female and male) maps for linkage group 13.** For LG13, the sex-average log likelihood is -411.8, the sex-specific (f,m) is -376.0, and the *P* value of the sex-specific heterogeneity test is highly significant at 9.6E-38 (FDR alpha value of 0.004). (EPS 1 MB)

Additional file 16: **The sex-average and sex-specific (female and male) maps for linkage group 14.** For LG14, the sex-average log likelihood is -388.8, the sex-specific (f,m) is -346.1, and the *P* value of the sex-specific heterogeneity test is highly significant at 1.1E-44 (FDR alpha value of 0.004). (EPS 1 MB)

Additional file 17: **Standardised female and male interval distances of LG3-LG6.** (PDF 353 KB)

Additional file 18: **Standardised female and male interval distances of LG7-LG10.** (PDF 345 KB)

Additional file 19: **Standardised female and male interval distances of LG11-LG14.** (PDF 345 KB)

Additional file 20: **Tests of segregation distortion for all intervals on the map.** Each interval was tested across each parent from the eight families where informative meiosis occurred using a G test. The family cross, G value, FDR alpha value and Significance are reported in addition to linkage map statistics. (XLSX 619 KB)

Additional file 21: **Estimates of the decline in linkage disequilibrium for individual linkage groups and the entire genome.** (XLSX 33 KB)

## Abbreviations

AFLP: Amplified fragment length polymorphism
CS1: Chitin synthase 1
FDR: False discovery rate
GO: Gene ontology
GWAS: Genome-wide association study
LD: Linkage disequilibrium
LG: Linkage group
LOD: Logarithm of odds
MAF: Minor allele frequency
MAS: Marker-assisted selection
MI: Mendelian inheritance
PFMG: *Pinctada fucata* mantle gene
QTL: Quantitative trait loci
QTN: Quantitative trait nucleotide
SNP: Single nucleotide polymorphism.
