# Supplementary figures and images for: A high-density SNP genetic linkage map for the silver-lipped pearl oyster, Pinctada maxima: a valuable resource for gene localisation and marker-assisted selection
Source: BMC Genomics. 2013 Nov 20;14(1):810. doi: 10.1186/1471-2164-14-810 (PMC4046678; doi:10.1186/1471-2164-14-810)

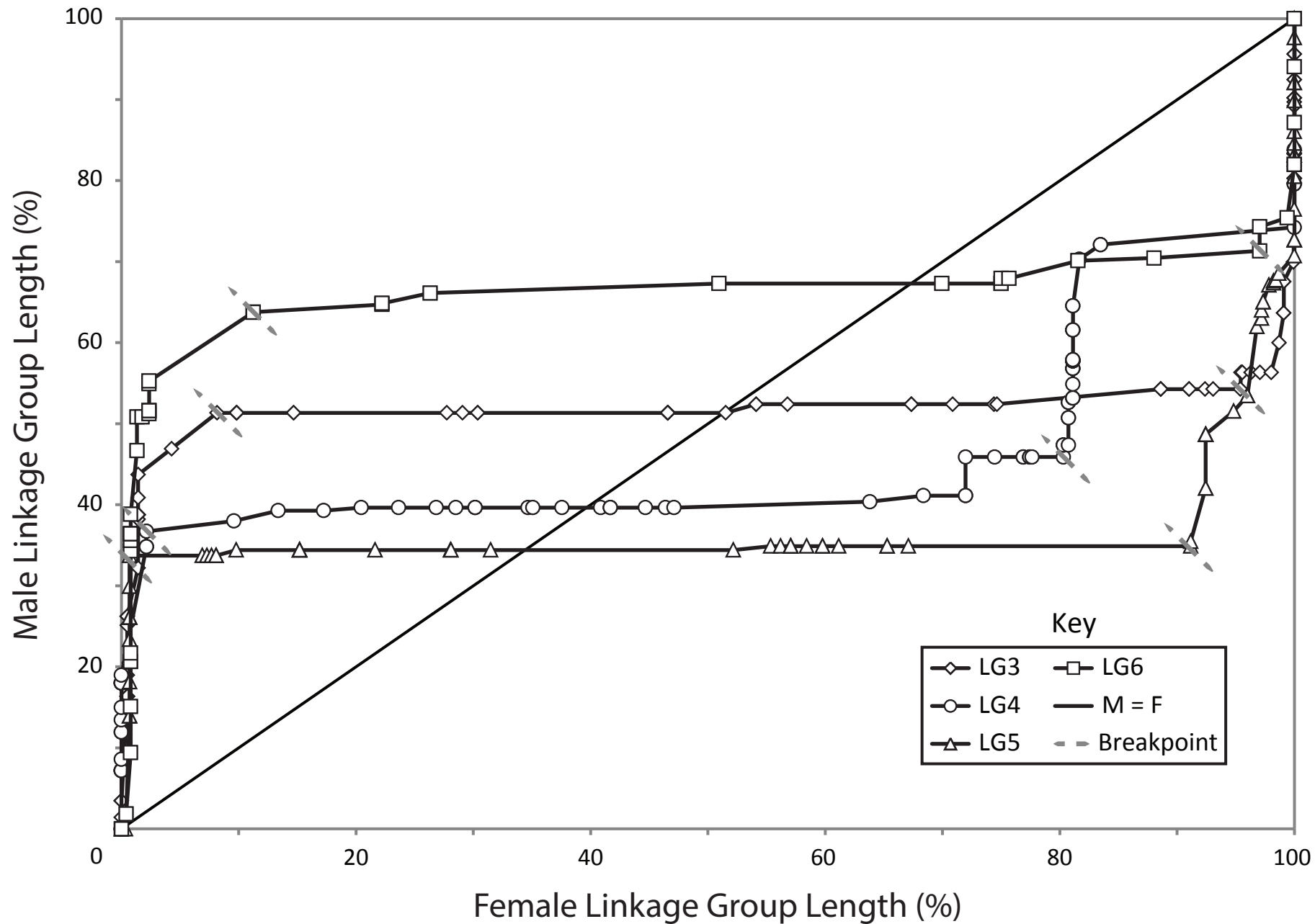

Supplement: Supplementary file 17 — Additional file 17: Standardised female and male interval distances of LG3-LG6. (PDF 353 KB) [file 12864_2013_5511_MOESM17_ESM.pdf]

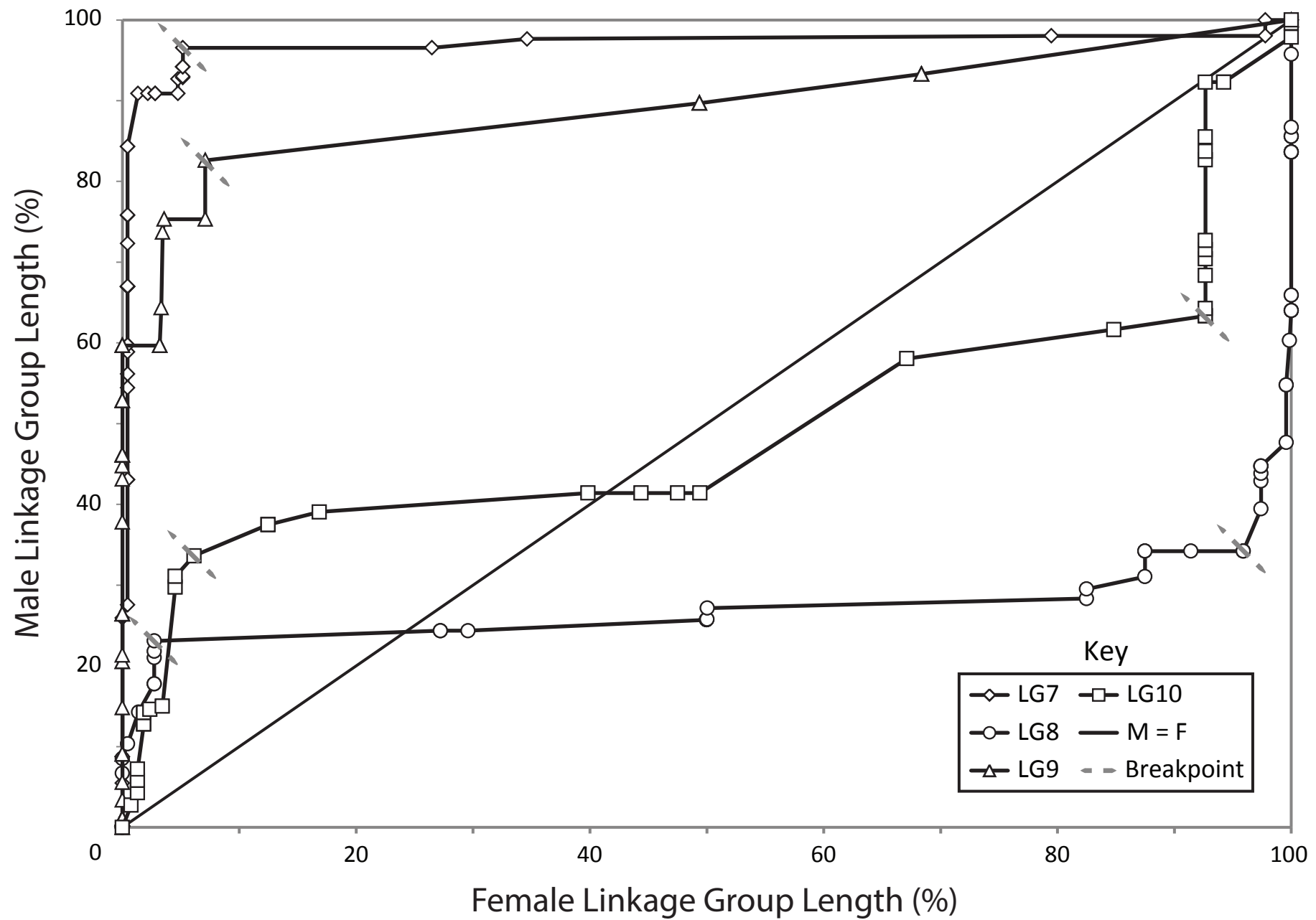

Supplement: Supplementary file 18 — Additional file 18: Standardised female and male interval distances of LG7-LG10. (PDF 345 KB) [file 12864_2013_5511_MOESM18_ESM.pdf]

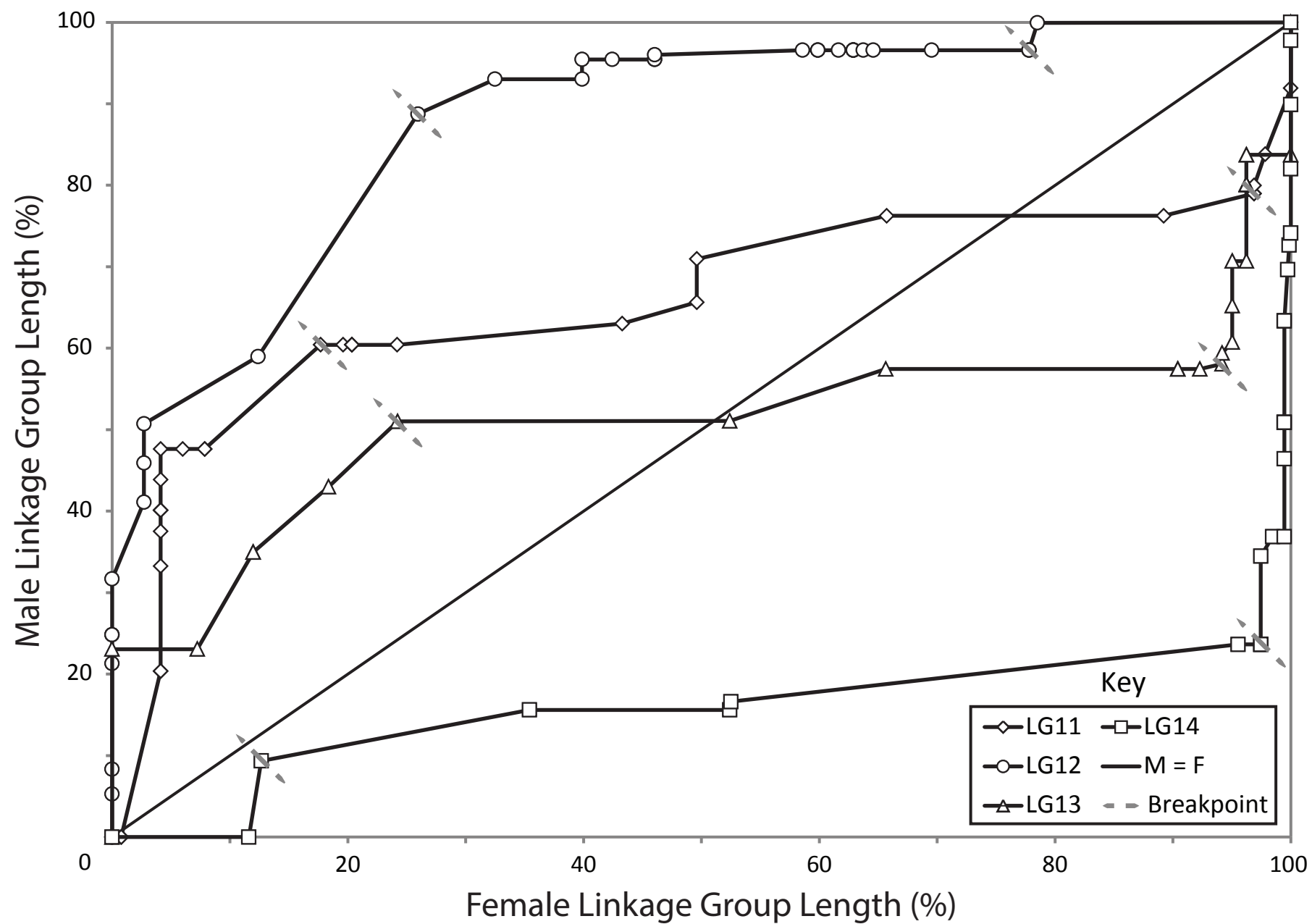

Supplement: Supplementary file 19 — Additional file 19: Standardised female and male interval distances of LG11-LG14. (PDF 345 KB) [file 12864_2013_5511_MOESM19_ESM.pdf]
